# Epigenetics in cardiovascular health, pathology, and development

**DOI:** 10.1080/15592294.2026.2722863

**Published:** 2026-09-01

**Authors:** Marta W. Szulik, Luis Hortells, Manuel Rosa-Garrido

**Affiliations:** Nora Eccles Harrison Cardiovascular Research & Training Institute, University of Utah, Salt Lake City, UT, USA; Cardiovascular Research Group, Department of Medical Biology, Faculty of Health Science, UiT-The Arctic University of Norway, Tromsø, Norway; Department of Cellular and Molecular Signalling, The Institute of Biomedicine and Biotechnology of Cantabria, CSIC-University of Cantabria, Santander, Spain

Cardiovascular diseases remain the leading cause of mortality worldwide [1]. Although substantial advances have been made in identifying the genetic determinants of cardiovascular disease, it has become increasingly clear that DNA sequence alone cannot explain the remarkable plasticity of cardiac development, adaptation, aging, and disease progression. Epigenetic regulation – including DNA methylation, histone modifications, chromatin accessibility, and non-coding RNA-mediated mechanisms – provides the dynamic interface through which environmental cues, metabolic state, and genetic background converge to shape different cardiac phenotypes. As technological advances continue to transform our ability to interrogate the epigenome, cardiovascular epigenetics has evolved from a descriptive discipline into one that is beginning to uncover mechanistic regulators and clinically actionable therapeutic targets.

The articles collected in this special issue illustrate this transition. Together, they demonstrate that epigenetic mechanisms are not isolated molecular events but highly interconnected regulatory systems governing cardiac development, physiological adaptation, pathological remodeling, and recovery. Rather than viewing individual epigenetic modifications independently, the studies presented here emphasize the coordinated interaction between chromatin architecture, DNA methylation, histone modifications, transcriptional regulation, and cellular metabolism in determining cardiovascular health and disease.

A major theme emerging from this collection is the growing power of integrative and multi-omics approaches to identify key regulatory nodes within complex cardiovascular networks. In this context, Lahue et al. [2] integrate genomic, methylome, and transcriptomic analyses to uncover regulatory hotspots that coordinate large-scale transcriptional responses during heart failure. This systems-level approach led to the identification of SERPINA3N as a previously unrecognized regulator of stress-induced cardiac remodeling. Importantly, this study moves beyond correlation by functionally validating SERPINA3N as a regulator of cardiomyocyte hypertrophy, illustrating how integrated omics can uncover mechanistic drivers of disease. In another study, Lahue et al. [3] mapped genome-wide DNA methylation changes across the Hybrid Mouse Diversity Panel (HMDP) to demonstrate that methylation patterns not only accompany heart failure progression but can predict susceptibility and identify therapeutically relevant loci. These findings illustrate how combining genetics, epigenetics, transcriptomics, and functional validation can bridge the longstanding gap between association studies and causal biology, providing a roadmap for future precision cardiovascular medicine.

Another recurring theme is the importance of chromatin organization as an active determinant of cardiac cell identity and disease progression. Beyond DNA and histone modifications, chromatin structure itself has emerged as a dynamic regulator of transcriptional programs. Using single-nucleus transcriptomic datasets, Geng et al. [4] demonstrate that the expression of chromatin structural genes effectively distinguishes pathological cardiomyocyte and fibroblast populations. Their identification of HMGN3 as a regulator of chromatin architecture and cardiomyocyte survival further highlights the functional importance of higher-order genome organization in pathological cardiac remodeling. These findings reinforce the emerging concept that alterations in higher-order genome organization are integral components of cardiovascular pathology rather than secondary consequences of disease. This concept is echoed by the work by Hickenlooper et al. [5], who investigated histone H4K20 methylation and its associated methyltransferases and demethylases in experimental models and human heart failure. Although no global changes in H4K20 dimethylation were observed, the authors identified a dynamic regulation of enzymes responsible for establishing this mark, suggesting that epigenetic remodeling occurs at the specific genomic loci rather than through widespread changes in histone methylation. Together, these studies illustrate the complexity of chromatin regulation, where higher-order genome organization and locus-specific histone modifications cooperate to shape transcriptional programs that initiate cardiac adaptation and disease.

Perhaps one of the most exciting conceptual advances represented in this collection is the growing integration of metabolism and epigenetics. In their comprehensive review, Konieczny et al. [6] trace the remarkable evolution of lactate from a metabolite once regarded simply as a byproduct of anaerobic glycolysis to an active signaling molecule and epigenetic regulator through protein lactylation. This expanding field illustrates how metabolic state can directly influence chromatin regulation and transcriptional programs, providing a mechanistic framework linking exercise, inflammation, fibrosis, vascular remodeling, and heart failure.

The concept that metabolic perturbations leave durable epigenetic consequences is further extended by the elegant translational study of Roth et al. [7], who investigated myocardial recovery following mechanical unloading in both a reversible mouse model of pressure overload and patients undergoing left ventricular assist device (LVAD) therapy. Although ventricular function and cardiac hypertrophy improved following unloading, the authors demonstrate that recovery was accompanied by persistent repression of mitochondrial metabolic programs together with sustained upregulation of multiple transcriptional and epigenetic regulators, including BRD4, HDAC4, SMARCA2, and NCOR2. These findings challenge the traditional assumption that simply removing pathological stress is sufficient to restore the myocardium to its pre-disease state. Instead, they suggest that cardiomyocytes enter a distinct recovered phenotype characterized by persistent alterations in transcriptional regulation and energy metabolism. This concept is thoughtfully placed into a broader biological context by Constantino et al. [8], whose accompanying commentary introduces the notion of an ‘epigenetic scar’ that persists despite normalization of mechanical stress. By drawing attention to enduring changes in mitochondrial gene expression and chromatin-modifying enzymes, the commentary proposes that incomplete myocardial recovery reflects not simply residual structural remodeling, but also long-lasting transcriptional memory embedded within the epigenome. This perspective raises an important therapeutic question for the field: whether future interventions should aim not only to halt pathological remodeling but also to actively reprogram persistent epigenetic states that continue to constrain myocardial recovery. Together, the work by Roth et al. [7] and the accompanying perspective by Constantino [8] shift the discussion of heart failure beyond disease progression toward mechanisms governing cardiac recovery, resilience, and reversibility.

While heart failure constitutes a major focus of this collection, the included studies also emphasize that epigenetic regulation contributes broadly across the cardiovascular disease spectrum. Martinis et al. [9] expand this perspective by investigating genome-wide DNA methylation patterns associated with essential hypertension in African Brazilian Quilombo populations, an understudied group with a disproportionately high prevalence of hypertension. Their identification of differentially methylated CpG sites and genomic regions associated with vascular biology, inflammation, renal function, salt homeostasis, and blood pressure regulation highlights DNA methylation as both a potential biomarker and a contributor to disease susceptibility. Importantly, this study also highlights the value of performing epigenetic investigations in genetically diverse and historically underrepresented populations. As precision cardiovascular medicine continues to evolve, understanding how ancestry, environmental exposures, and epigenetic regulation intersect will be essential for translating epigenomic discoveries into diagnostic and therapeutic strategies. The translational potential of cardiovascular epigenetics is further illustrated by Andersen et al. [10], who demonstrate the utility of DNA methylation biomarkers in patients presenting with acute coronary syndrome. Using methylation-derived biomarkers of chronic heavy alcohol consumption and smoking exposure, the authors show that an epigenetic measure of heavy alcohol consumption independently predicts subsequent mortality, whereas smoking-associated methylation primarily reflects the burden of coronary artery obstruction. These findings highlight how epigenetic biomarkers can capture cumulative environmental exposures that may not be adequately assessed through conventional clinical evaluation, illustrating their potential value for risk stratification and personalized cardiovascular care. When considered alongside the population-based methylation analyses by Martinis et al. [9], these studies highlight the expanding clinical relevance of epigenetics, from identifying disease susceptibility to improving prognostic assessment and informing precision medicine strategies.

Another emerging direction emphasized in this collection is the growing appreciation that epigenetic regulation does not occur in isolation but is closely integrated with endocrine and circadian signaling. In their comprehensive review, Akinborewa et al. [11], discuss the epigenetic activity of the glucocorticoid receptor (GR), emphasizing its central role in regulating cardiomyocyte hypertrophy, calcium handling, and metabolic homeostasis through both genomic and non-genomic mechanisms. Importantly, the authors highlight that GR signaling is essential not only for maintaining adult cardiac function but also for fetal heart development, reinforcing the concept that developmental epigenetic programs continue to influence cardiovascular physiology throughout life. Their discussion of the interaction between epigenetic regulation and circadian rhythms further introduces the intriguing possibility that chronobiology may become an important determinant of future epigenetic therapies, where the timing of glucocorticoid administration could optimize therapeutic efficacy while minimizing adverse effects. This concept is further strengthened by Arrieta et al. [12], who demonstrate that environmental cues including adrenergic stimulation, hypoxia, and paracrine signaling dynamically regulate the circadian expression of the sarcomeric genes *Tcap* and *Myl2* during postnatal cardiac maturation. Their findings identify Titin-cap (TCAP) as a previously unrecognized mediator linking circadian clock activity with cardiomyocyte growth, maturation, and adaptation to environmental stress. Notably, the study reveals that environmental perturbations remodel circadian transcriptional programs through BMAL1-dependent mechanisms while influencing hypertrophic responses and structural gene expression. By integrating circadian biology, developmental maturation, and environmental sensing, this work extends the concept that the cardiac clock is not simply a passive timing mechanism but an active regulator of myocardial adaptation to physiological and pathological stimuli.

Collectively, these studies redefine the conceptual boundaries of cardiovascular epigenetics. Rather than focusing exclusively on chromatin regulation within diseased cardiomyocytes, they demonstrate that epigenetic mechanisms integrate metabolism, environmental exposures, transcriptional memory, and population diversity into a unified regulatory framework. This emerging paradigm reinforces the view that epigenetic regulation is not merely a downstream consequence of cardiovascular disease but can also precede disease onset, predisposing the myocardium to pathological remodeling and acting as a dynamic and potentially reversible determinant of disease susceptibility, progression, and recovery. Equally significant, these studies reflect an important evolution in methodology. The field is rapidly progressing from bulk tissue analyses toward single-cell and single-nucleus transcriptomics, integrative multi-omics, chromatin accessibility profiling, and functional validation in experimental models. These approaches provide increasingly sophisticated tools for delineating the remarkable cellular heterogeneity of the heart while enabling identification of disease-driving pathways with unprecedented resolution. Importantly, many of the studies move beyond association by experimentally validating candidate regulators, thereby strengthening the translational relevance of their findings.

Despite this progress, significant challenges remain. Much of our current understanding still derives from experimental models, highlighting the emerging need for expanded validation in diverse human populations and across different cardiovascular diseases. Integrating epigenomic information across multiple regulatory layers – including DNA methylation, histone modifications, chromatin accessibility, non-coding RNAs, and three-dimensional genome organization – remains an important objective. Thus, distinguishing adaptive from maladaptive epigenetic remodeling, defining the temporal dynamics of epigenetic remodeling during disease progression and recovery, and understanding cell-type-specific regulation will be essential for translating epigenetic discoveries into precision cardiovascular medicine.
